# A Weather-Driven Model for Predicting Infections of Grapevines by Sporangia of *Plasmopara viticola*

**DOI:** 10.3389/fpls.2021.636607

**Published:** 2021-03-09

**Authors:** Chiara Brischetto, Federica Bove, Giorgia Fedele, Vittorio Rossi

**Affiliations:** ^1^Department of Sustainable Crop Production (DI.PRO.VE.S.), Università Cattolica del Sacro Cuore, Piacenza, Italy; ^2^Horta Srl, Piacenza, Italy

**Keywords:** downy mildew, secondary infections, weather-based model, disease prediction, model evaluation, modeling

## Abstract

A mechanistic model was developed to predict secondary infections of *Plasmopara viticola* and their severity as influenced by environmental conditions; the model incorporates the processes of sporangia production and survival on downy mildew (DM) lesions, dispersal and deposition, and infection. The model was evaluated against observed data (collected in a 3-year vineyard) for its accuracy to predict periods with no sporangia (i.e., for negative prognosis) or with peaks of sporangia, so that growers can identify periods with no/low risk or high risk. The model increased the probability to correctly predict no sporangia [P(P−O−) = 0.67] by two times compared to the prior probability, with fewer than 3% of the total sporangia found in the vineyard being sampled when not predicted by the model. The model also correctly predicted peaks of sporangia, with only 1 of 40 peaks unpredicted. When evaluated for the negative prognosis of infection periods, the model showed a posterior probability for infection not to occur when not predicted P(P−O−) = 0.87 with only 9 of 108 real infections not predicted; these unpredicted infections were mild, accounting for only 4.4% of the total DM lesions observed in the vineyard. In conclusion, the model was able to identify periods in which the DM risk was nil or very low. It may, therefore, help growers avoid fungicide sprays when not needed and lengthen the interval between two sprays, i.e., it will help growers move from calendar-based to risk-based fungicide schedules for the control of *P. viticola* in vineyards.

## Introduction

Downy mildew (DM) is an important disease of grapevines, and much research has focused on its causal agent, the oomycete *Plasmopara viticola* (Berk. & M. A. Curtis) Berl. & De Toni. The life cycle of *P. viticola* consists of sexual and asexual cycles that occur throughout the grapevine growing season and that are driven by oospores and sporangia, respectively.

In the last century, researchers assumed that infection by oospores was only important early in the grapevine vegetative season. They attributed the explosive development of the disease to the sporangia, which originated from asexual reproduction and which were assumed to migrate over distances in a short time. In the 2000s, researchers investigated the previous assumptions regarding the pathogen’s epidemiology (Gobbin et al., 2003) and especially the qualitative and quantitative contribution of oosporic vs. clonal (asexual) infections (Gessler et al., 2003; Gobbin et al., 2005). Combining epidemiological and population genetics data, a broader perspective of the disease dynamics was finally obtained (Rumbou and Gessler, 2004; Gobbin et al., 2006). According to the new perspective, the role of secondary (asexual) cycles for the epidemic development had been overestimated in the past. Nevertheless, secondary cycles may be locally important, and growers pay great attention to their control with repeated, calendar-based fungicide applications (Gessler et al., 2011).

Secondary disease cycles involve the processes of sporulation, dispersal, and infection. Within 5–10 days after the infection, depending on weather conditions (Blaeser and Weltzien, 1979; Lafon and Clerjeau, 1988; Schruft and Kassemeyer, 1999), the pathogen emerges from stomata and forms sporangiophores and sporangia on DM lesions. Sporulation becomes visible as a dense, raised, white-cottony mildew on the abaxial surface of leaves, on green shoots, and on young berries. Sporangia are dispersed by rain (Kast, 1997) and wind (Diaz et al., 1997, 1998; Fernandez-Gonzales et al., 2009; Magyar et al., 2009; Caffi et al., 2013a), and are deposited on the host tissue. When host surfaces are moistened by rainfall or dew, sporangia release six to eight clonal zoospores (Lalancette et al., 1987) as secondary inoculum, which are able to further infect all green tissues of the vines.

Some mathematical models have been developed to provide short-term and field-scale predictions of DM epidemics resulting from infections caused by *P. viticola* sporangia in Switzerland, France, Austria, Germany, and Italy (Blaise and Gessler, 1990; Hill, 1990; Magarey et al., 1991; Magnien et al., 1991; Orlandini et al., 1993; Ellis et al., 1994; Blaise et al., 1999; Leroy et al., 2013). All of these models have been developed using a common database of previous publications and are based, more or less explicitly, on HLIR models, in which H, L, I, and R represent healthy sites, latent sites, infectious sites, and removed sites, respectively (Zadoks, 1971; Madden et al., 2007). In these models, the number of new lesions developing on the healthy tissue on any day depends on the following variables: (i) the amount of susceptible host tissue that is disease-free and can be infected; (ii) the number of spores that are available (i.e., spores that are produced, dispersed, and deposited on the host surface); and (iii) the infection efficiency of available spores. The number of available spores depends on the number of sporulating lesions and on the quantity of spores produced per unit of sporulating lesion (Zadoks, 1971; Zadoks and Schein, 1979). These models simulate the temporal dynamics of affected sites (i.e., L+I+R sites) in terms of disease severity or a disease index. Some of these models also enable the mobilization of available knowledge on the *P. viticola*-*Vitis vinifera* system and the exploration of the system’s behaviour under different environmental conditions, e.g., in different years and/or locations (Bove et al., 2020a,b).

The above-mentioned models have limitations when used for guiding fungicide applications to control DM. Because *P. viticola* can rapidly cause devastating epidemics, DM control aims to prevent the establishment of the disease in the vineyard by controlling primary infections (Caffi et al., 2009) and to prevent secondary spread as soon as the first seasonal DM lesions appear in the vineyard. A model that is able to predict the occurrence of favourable conditions for secondary infections may, therefore, be more useful than models that simulate disease progress over time. To date, however, such a model does not exist.

In the current study, a weather-driven, mechanistic model for predicting secondary infection by *P. viticola* was, therefore, developed. A preliminary version of the model was published in 2013 (Caffi et al., 2013b). In the current study, the latter model was updated with recent epidemiological findings and was validated with a 3-year data set collected in a vineyard.

## Materials and Methods

### Model Description

The model was designed to (i) produce warnings about the occurrence of environmental conditions favourable for secondary infection periods and (ii) assess the relative severity of the corresponding infections. The model was not intended to provide an assessment of the progeny/parent ratio, i.e., the number of DM daughter lesions produced per mother lesion in each infection period and, consequently, to simulate the DM progress curve in a vineyard; such a model was recently developed in another study (Bove et al., 2020a).

**Figure 1:**
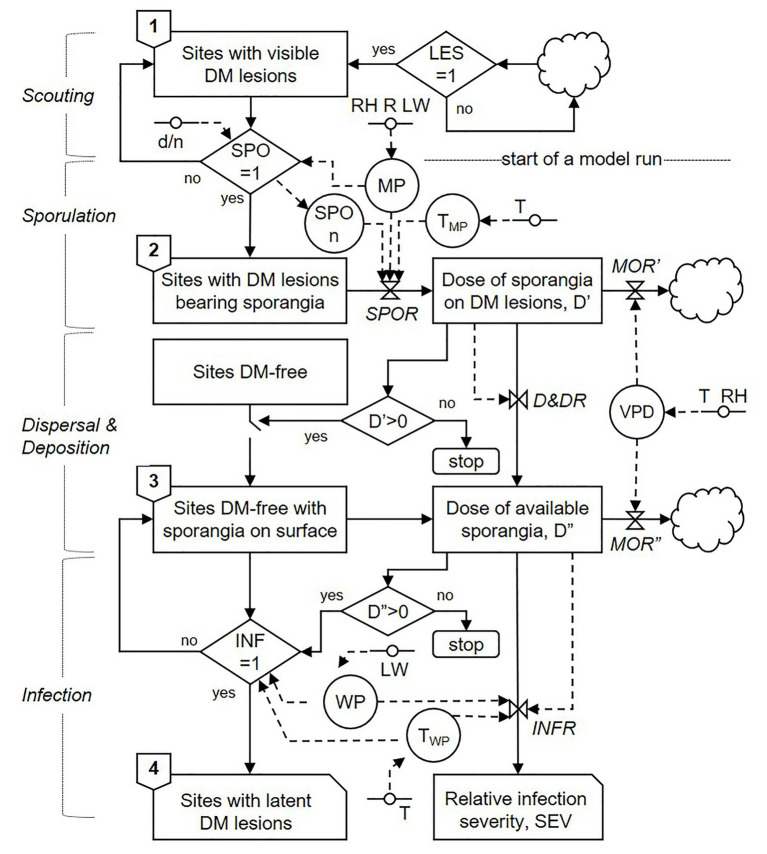
Relational diagram of the model predicting secondary infection cycles of *P. viticola*, drawn following the symbols of systems analysis as in Rossi et al. (2010). Boxes represent state variables; solid arrows represent flows that connect state variables; dashed arrows represent flows that connect driving variables to rates; circles represent parameters; rhombuses represent switches; clouds represent outgoing variables; valves represent rates; lines with a circle in the middle represent driving variables (acronyms of all of the elements of the model and their units are listed in Table 1). Lesion onset (LES), sporulation onset (SPO), and infection occurrence (INF) are dichotomic variables, with values = 1 when there are lesions, sporulation events, and infections, respectively, and values = 0 when there are no lesions, sporulation events, or infections, respectively.

The model begins when the first seasonal DM lesions appear in the vineyard, which are detected by scouting; scouting and other general processes of the model are indicated on the left side of Figure 1. Once DM lesions appear, the model assumes that lesions will be present all season long and that these lesions may produce sporangia under suitable conditions; that assumption is reasonable given that a single DM lesion can sporulate several times, and that spore production can continue after unfavourable periods (Kennelly et al., 2007; Caffi et al., 2013a). The first model step, therefore, consists of “sites with visible DM lesions” (Figure 1). The term “site” refers to a unit of host surface that can sustain a DM infection and potentially give raise to new infections, and the total number of sites is the host carrying capacity (Van der Plank, 1963). Sites can be either DM-free or occupied by a DM lesion, and the latter can either be a visible non-sporulating lesion or a sporulating lesion.

The model assesses the main biological and epidemiological processes involved in secondary infections by *P. viticola* and is organised into three compartments: (i) sporulation; (ii) dispersal and deposition of sporangia; and (iii) infection. A relational diagram of the model is shown in Figure 1; variables are listed in Table 1.

**Table 1:** Description of the stages in the system, state variables, switch variables, rates, intermediate variables, and external variables used in the relational diagram of the model presented in Figure 1.

Variable	Acronym	Unit
**Stages in the system**		
Sites with visible DM^1^ lesions	-	Dimensionless
Sites with DM lesions bearing sporangia	-	Dimensionless
Sites DM-free	-	Dimensionless
Sites DM-free with sporangia on surface	-	Dimensionless
Sites with latent DM lesions	-	Dimensionless
**State variables**		
Dose of sporangia on DM lesions	D’	Number
Dose of available sporangia for infection	D”	Number
Relative infection severity	SEV	Number
**Switch variables**		
Lesion onset	LES	0 (no lesions)/1(there are lesions)
Sporulation onset	SPO	0 (no sporulation)/1 (sporulation)
Infection occurrence	INF	0 (no infection)/1 (infection)
**Rate variables**		
Sporulation rate	*SPOR*	0–1
Mortality rate of sporangia on DM lesions	*MOR’*	0–1
Dispersal and deposition rate	*D&DR*	=1
Mortality rate of detached sporangia	*MOR”*	0–1
Infection rate	*INFR*	0–1
**Intermediate variables**		
Moisture duration (or moist period)	MP	N. of hours
Number of sporulation events	SPOn	Number
Average temperature of MP	T_MP_	°C
Wetness duration (or wet period)	WP	N. of hours
Average temperature of WP	T_WP_	°C
Vapour pressure deficit	VPD	kPa
**External variables**		
Day/night	-	0 (daylight)/1 (night time)
Air temperature	T	°C
Relative humidity	RH	%
Rain	R	mm
Leaf wetness	LW	min

1 DM, downy mildew.

#### Sporulation Compartment

Sites with DM lesions produce sporangiophores and sporangia when weather conditions are favourable; the occurrence of favourable conditions is regulated by the switch SPO (Figure 1). If SPO = 1, then lesions advance the second model step, i.e., “sites with DM lesions bearing sporangia”; if SPO = 0, in contrast, lesions remain in the first model step (i.e., they remain non-sporulating). Because *P. viticola* sporangia emerge from the lesion surface and produce sporangia in the dark when there is moisture and when temperature is >10°C (Blaeser and Weltzien, 1979; Gessler et al., 2011), the model sets SPO = 1 when the night-time moist period (MP) is ≥3 h and the temperature of the moist period (T_MP_) is between 10 and 30°C (Lalancette et al., 1988b); an hour is considered moist when the relative humidity (RH) is ≥80%, rain (R) is >0 mm, or leaf wetness (LW) is >30 min (Caffi et al., 2013a). Once sporulation is triggered (i.e., SPO = 1), the model initiates a “run.” Therefore, the number of model runs in a season is equivalent to the number of days that SPO = 1 in a season; a model run can result or fail to result in a DM infection (Figure 1).

**Figure 2:**
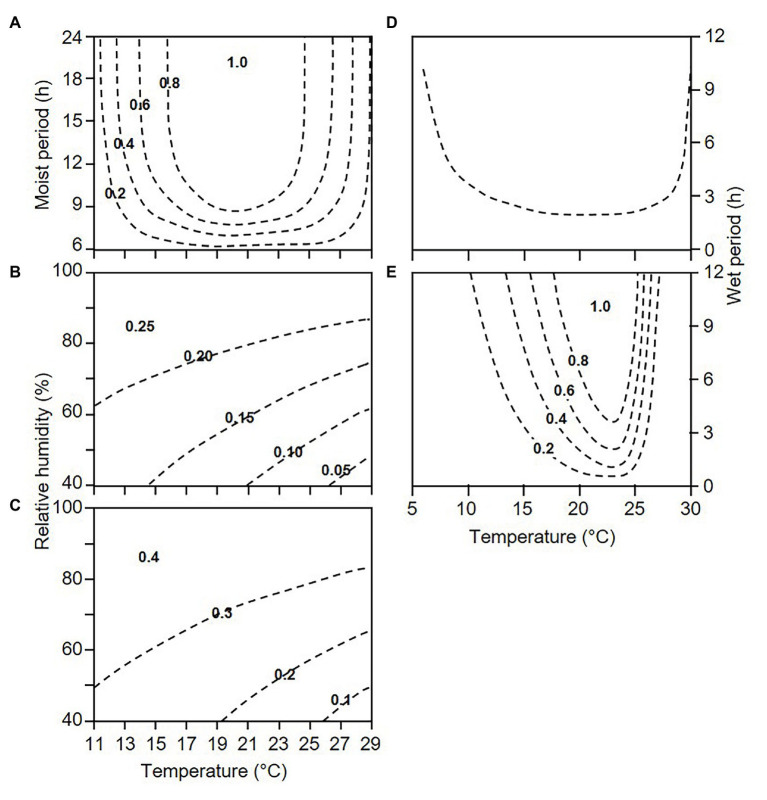
Contour plots showing the relationships between temperature and wetness duration or relative humidity and the following elements used in the model (see Figure 1): **(A)** sporulation rate, *SPOR*; **(B)** mortality rate of sporangia attached to sporangiophores on downy mildew (DM) lesions, *MOR’*; **(C)** mortality rate of sporangia detached from sporangiophores, *MOR”*; **(D)** minimal requirements for infection; and **(E)** infection rate, *INF*. Equations were obtained from the following sources: **(A)** the model of Lalancette et al. (1988a) for describing the sporulation of *P. viticola* on grape leaves, in which the numbers of sporangia per cm^2^ of leaf were rescaled by dividing by the number at 20°C and 24 h of a wet period; **(B,C)** the models relating temperature and relative humidity, expressed as vapour pressure deficit, as used by Brischetto et al. (2020) for the sporangia attached to or detached from sporangiophores; **(D)** the model of Magarey et al. (2005) with the following parameters estimated from data of Blaeser and Weltzien (1979) and Caffi et al. (2016), with *R*^2^ = 0.87 for estimated vs. observed data: shortest wetness duration at optimal temperature = 2 h; minimal, optimal, and maximal temperature for infection = 4.0, 21.0, and 30.2°C, respectively; and **(E)** the model of Caffi et al. (2016) for infection of grape leaves by *P. viticola*.

When lesions begin producing sporangia, they continue sporulating as long as weather conditions remain favourable (as defined before); this is a “sporulation period” in the model. During each sporulation period, the relative “dose of sporangia on DM lesions” (D’) is calculated with a sporulation rate, *SPOR*, by using the equation shown in Figure 2A, which is modulated by the number of sporulation events (SPOn) by using the equation of Kennelly et al. (2007). As the sporulation period ends, the dose of sporangia progressively diminishes because the sporangia still attached to sporangiophores may die under unfavourable conditions of temperature and relative humidity (Blaeser and Weltzien, 1979; Kast and Stark-Urnau, 1999). The model calculates the reduction of sporangial dose through a mortality rate *MOR’*, which is a function of the vapour pressure deficit (VPD), as shown in Figure 2B.

#### Spore Dispersal and Deposition Compartment

The next model step consists of DM-free sites on which the sporangia deposit after being detached from sporangiophores on sporulating DM lesions and dispersed into the air (Figure 1). Because *P. viticola* sporangia become airborne under a wide range of environmental conditions (Caffi et al., 2013a) and because they are normally present in the air of DM-affected vineyards (Diaz et al., 1997, 1998; Albelda et al., 2005; Fernandez-Gonzalez et al., 2009; Magyar et al., 2009; Fernández-González et al., 2011, 2019; Martínez-Bracero et al., 2019; Rodríguez et al., 2020), the model assumes that whenever there are sporangia on DM lesions, sporangia may detach, disperse, and be deposited on the host surface, i.e., when D’ > 0; if D’ = 0, there are no viable sporangia, and the model run ends.

The relative “dose of available sporangia” on DM-free sites (D”) is calculated by a dispersal and deposition rate, *D&DR*. The model assumes that all of the sporangia on DM lesions have the same probability of becoming airborne and being deposited on the host surface; therefore, *D&DR* = 1. This dose of sporangia, however, progressively diminishes because the sporangia detached from sporangiophores can die under unfavourable conditions (Blaeser and Weltzien, 1979). In the model, the reduction of sporangial dose is calculated through a mortality rate *MOR”,* as a function of VPD as shown in Figure 2C.

#### Infection Compartment

The last model step involves the sites that become latently infected (i.e., DM lesions that are not yet visible because the incubation period is not over; Figure 1). These sites enter into this stage when there are viable sporangia on DM-free sites, i.e., when D” > 0 (if D” = 0, the model run ends) and there are favourable conditions for infection. The presence of favourable conditions is regulated by the switch INF. If INF = 1, then lesions advance to the fourth model step: “sites with latent DM lesions”; otherwise INF = 0 and lesions remain in the third model step (i.e., sites remain DM-free with sporangia on their surface).

For infection to occur, sporangia may release zoospores into water, and zoospores may swim to stomata, encyst, and produce germ tubes that penetrate the stomatal opening (Emmett et al., 1992). In the model, the time required for completing these processes is an “infection period,” i.e., a period of hours with uninterrupted leaf wetness or with leaf wetness that is interrupted for a maximum 1 h (WP); in the model, INF = 1 when the WP at the registered temperature (T_WP_) is longer than the minimum required for that temperature based on Figure 2D.

The relative infection severity of each infection period is finally calculated by an infection rate, *INFR*, which depends on WP and T_WP_, as shown in Figure 2E.

**Figure 3:**
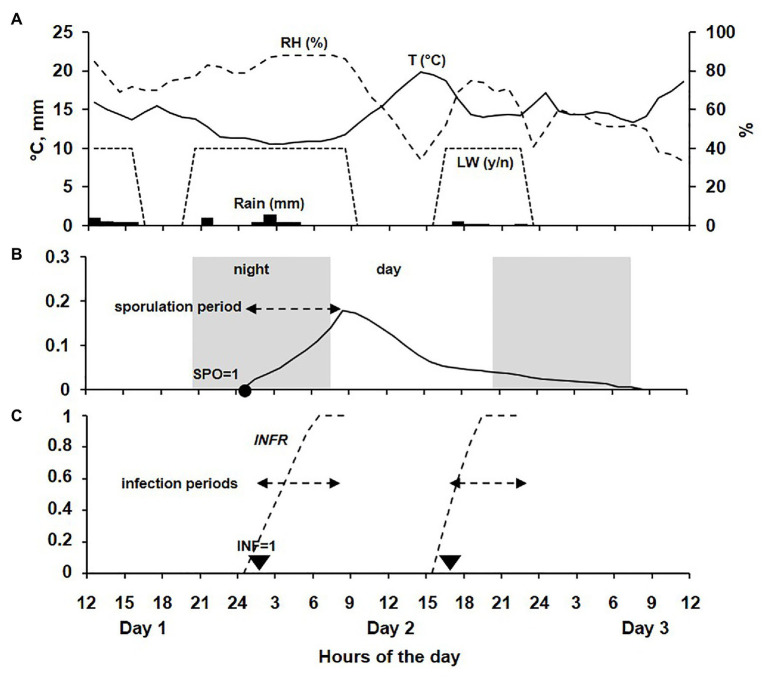
Example of model output for a 48-h period across 3 days. **(A)** Data for temperature (T, °C, line), relative humidity (RH, %, dotted line), rain (R, mm; bars), and leaf wetness (LW, as presence or absence, dotted trapezoid). **(B)** Sporulation rate on DM lesions during a sporulation period beginning in the dark at the time when SPO = 1. **(C)** Infection rate during an infection period beginning at the time when INF = 1.

An example of the model output is shown in Figure 3.

### Model Evaluation

Independent data (i.e., data not used in model development) collected during three growing seasons (2015–2017) from an experimental vineyard were used to validate the model’s ability to predict (i) the presence of sporangia, (ii) the occurrence of *P. viticola* infections on grape leaves, and (iii) the relative infection severity.

#### Experimental Vineyard

Data were collected in the vineyard described by Brischetto et al. (2020). In brief, the vineyard was located on the campus of Università Cattolica del Sacro Cuore, Piacenza (northern Italy, 45° 2'N, 9° 43'E). It was planted in 2006 with *V. vinifera* cultivar Barbera, which is susceptible to DM (Rossi and Caffi, 2007). Vines were spaced 1.1 m in the row and 1.3 m between rows, and were trained on a single Guyot system. No fungicides were used for the duration of the study. A standard meteorological station (iMetos®, Pessl Instruments, Austria) located in the experimental vineyard recorded hourly air temperature (T, °C), relative humidity (RH, %), rainfall (R, mm), leaf wetness (LW, min), and wind speed (m/s).

To ensure the presence of *P. viticola* in the vineyard, six vine shoots regularly located along two adjacent vine rows were inoculated with a suspension of *P. viticola* sporangia on 14 May 2015, 19 May 2016, and 18 May 2017. The inoculation method was described by Brischetto et al. (2020).

#### Sampling of Airborne Sporangia

A volumetric spore sampler (VPPS-2000 Lanzoni, Bologna, Italy) placed at 1.5 m above the soil surface and between the two artificially inoculated rows was used to sample the airborne sporangia of *P. viticola* from 11 May to 14 September 2015 (127 days), 11 May to 30 September 2016 (143 days), and 18 May to 28 September 2017 (134 days). The air was aspirated through the spore sampler at a flow rate of 10 litres per minute. The airflow was directed toward a Melinex transparent tape (34 cm long and 1.4 cm wide) mounted on a cylinder that rotated at 2 mm hr^−1^. The tape was coated with a silicone film to allow the deposition of spores and was replaced every 7 days. The collected tape was cut into seven 48-mm segments, each of which was mounted in glycerin jelly with fucsina (Lanzoni s.r.l., Bologna) on a microscope slide and was protected with a cover glass. Each segment corresponded to 1 sampling day; the *i*^th^ sampling day began at 10:00 h of the day *i*^th-1^ and ended at 09:00 h of the *i*^th^ day. *P. viticola* sporangia m^−3^ of air on each sampling day (total number of sporangia, SPT, m^−3^ air day^−1^) were counted using a microscope (20x magnification; Brischetto et al., 2020).

#### Assessment of DM Infection

The occurrence of infection was assessed by counting the DM lesions formed on grape leaves that were exposed to sporangia in the vineyard and that were then incubated in the laboratory under optimal conditions for infection, as explained in Brischetto et al. (2020). In brief, at 2- or 3-day intervals at 10:00 AM, 20 random leaves without visible DM symptoms were collected in the two rows of vines where *P. viticola* had been inoculated and the spore sampler operated. Over the 3 years of the study, leaves were collected on 108 dates (35, 37, and 36 dates in 2015, 2016, and 2017, respectively). After each sampling, 20 leaf fragments of approximately 8 cm^2^ were excised (1 fragment per leaf) with a scissors. Leaf fragments were placed abaxial side up in Petri dishes on wet blotting-paper, and were sprayed with sterile-distilled water so as to form a uniform film of water. After they were sealed with Parafilm to maintain a saturated atmosphere, the Petri dishes were incubated at 23°C with a 12-h photoperiod. After 24 h (which is sufficient for the sporangia to cause infection; Unger et al., 2007), leaf fragment surfaces were dried with sterile filter paper (OIV, 2009) and incubated again under the same conditions.

Leaf fragments were observed daily for 4 days with a stereomicroscope at 10x magnification to detect DM lesions. These DM lesions were considered to have occurred in the field and were assumed to be in the latency stage at the time of leaf collection, because the incubation period at 23°C is 4 days (Goidànich, 1964). The number of DM lesions per each sampling day (NLL) was expressed per leaf fragment, i.e., per 8 cm^2^ of leaf.

### Data Analysis

A Bayesian analysis of SPT values was used to evaluate the model ability to determine the periods in which the sporangia of *P. viticola* were present in the vineyard (Yuen and Hughes, 2002). For this purpose, every day of the study period was categorised as a day on which significant numbers of sporangia were observed (O+) or were not observed (O−); sporangia were considered to be present in significant numbers when there were >0.2 sporangia m^−3^ air day^−1^, with 0.2 being the 25^th^ percentile of the SPT distribution observed during the study. The peaks of sporangia were also considered, being days with >87.9 sporangia m^−3^ air, with 87.9 being the 90^th^ percentile of the SPT distribution. Similarly, every day was categorised as a day on which sporangia were predicted to be present (P+) or absent (P−) based on whether the dose of available sporangia D” was higher than or equal to zero, respectively. All possible combinations of predicted vs. observed days with or without sporangia were organised in a 2 × 2 contingency table, where P−O− (sporangia were not predicted to be present and no significant numbers of sporangia were sampled) and P+O+ (sporangia were predicted to be present and they were sampled in significant numbers) were the correct estimates, while P+O− and P−O+ were the incorrect estimates. To assess the practical advantages of using the model, the posterior probabilities that a predicted sporulation period resulted or did not result in a real one were determined as P(O+P+) and P(O−P−), respectively (Madden et al., 2007), and were compared with the corresponding prior probabilities, P(O+) and P(O−), respectively.

Data on the number of DM lesions on leaves (NLL) sampled from the vineyard on the 108 sampling days of the study period were used to evaluate the model ability to predict the occurrence of *P. viticola* infection on grape leaves, as well as the relative severity of these infections. Each day was categorised as a day on which *P. viticola* infection was predicted or not based on whether the switch variable INF was equal to one or zero, respectively (see example in Table 2), and the relative severity of infection (SEV) predicted by the model was noted. Afterward, days on which there were latent infections, i.e., infections not yet visible because the incubation period was not over, were calculated. For this purpose, the daily percentage of incubation progress was calculated for each infection as a function of temperature and relative humidity by using the equation of Orlandini et al. (2008); the incubation was considered not finished when the incubation progress was <100%. In each day, the number of infections that were still in incubation and the corresponding cumulative values of SEV were calculated (Table 2). These cumulative severities of the days in which leaves were sampled from the vineyards were then used for comparison with real values.

**Table 2:** Example of calculations for the comparison of the *Plasmopara viticola* infections predicted by the model and observed on grape leaves from the vineyard.

Day^1^	Model output						Reality		Model vs. reality
	Occurrence (and severity) of infection^2^	Incubation progress (%) for infection on day^3^			N. of infections (and cumulated severity)^4^	Prediction of infection^5^	N. DM lesions on leaves^6^	Observation of infection^7^	
		i+4	i+5	i+6					
i	0				0				
i+1	0				0				
i+2	0				0	P−	0	O−	P−O−
i+3	0				0				
i+4	1(0.137)	18			1(0.137)				
i+5	1(0.298)	38	20		2 (0.435)	P+	0	O−	P+O−
i+6	1(0.281)	58	40	20	3 (0.716)				
i+7	0	79	61	40	3 (0.716)				
i+8	0	98	80	60	3(0.716)				
i+9	0		91	70	2(0.579)	P+	1.7	O+	P+O+
i+10	0			83	1(0.281)				
i+11	0			96	1(0.281)				
i+12	0				0	P−	0.1	O+	P−O+
i+13	0				0				

1 Day of model simulation.

2 Infection occurrence (=1) or not (=0) on day *i*, and its severity (in brackets) based on model output.

3 Calculation of incubation progress begins on each day “i” in which infection is predicted; the percentage of incubation progress on each day is calculated as a function of temperature and relative humidity by using the equation of Orlandini et al. (2008).

4 Number of infections that are incubating on each day and corresponding cumulative values of severity.

5 Days are categorised as P+ (days on which the model predicts that there are latent infections) or P− (days on which the model predicts that there are no latent infections).

6 Average number of downy mildew (DM) lesions per leaf piece (8 cm^2^) on asymptomatic leaves collected in the vineyard.

7 Days are categorized as O+ (days on which the leaves hold latent infections) or O− (days on which leaves are infection free).

For comparison, sampling days on which the asymptomatic leaves taken from the vineyard showed DM lesions after incubation in the laboratory (i.e., NLL > 0) were considered as days on which leaves held latent infections (O+); similarly, days on which the field collected leaves did not show any *P. viticola* symptom after incubation were considered as days on which leaves were infection free (O−; see example in Table 2).

In a first analysis, a receiver operating characteristic (ROC) curve (Hanley, 2005) was created, in which cumulative values of SEV were considered as possible predictors of *P. viticola* infection of the grape leaves sampled from the vineyard. The ROC curve displayed the proportion of cases (leaf samples) correctly classified as carrying a latent infection, i.e., true positive proportion (TPP, or sensitivity), vs. the proportion of cases wrongly classified as non-infected, i.e., false negative proportion (FNP, or 1-specificity), across a range of cut-offs of cumulative SEV. The ROC curve represents the trade-off between sensitivity and specificity and was used to identify the best cut-off, corresponding to the highest overall accuracy of the test, i.e., the highest ratio between the number of cases assigned to the correct category and the number of cases that actually belonged to that category (Zweig and Campbell, 1993). The overall accuracy was expressed as the area under the ROC curve (AUROC), and its 95% confidence interval was calculated. The larger the AUROC (in the range 0.5–1), the better the performance of the binary classifier system in distinguishing between the two groups (infection/no infection). The value of *p* was calculated as the probability that the AUROC is different from the null hypothesis, i.e., that AUROC = 0.5 (the ROC curve coincides with the 1st diagonal) and that the variable under study does not distinguish between the two groups. The optimal cut-off point was determined by calculating the square of the distance between the point (0 and 1) on the upper left hand corner of the ROC space and any point on the ROC curve, as *d*^2^ = (1-sensitivity)^2^ + (1-specificty)^2^ (Kumar and Indrayan, 2011).

In a second analysis, sampling days were categorised as days on which the model predicted there were latent infections (P+) and days on which no latent infections were predicted (P−; see the example of Table 2), by using the best cut-off value obtained from the ROC curve. Model predictions (either P+ or P−) were compared with the observations in the vineyard (that also were either O+ or O−). Data were organized in a 2 × 2 contingency table and were subjected to a Bayesian analysis (Yuen and Hughes, 2002) as described for sporulation. In this case, P−O− defines days on which infections were not predicted to be present and no DM lesions appeared on field collected leaves, and P+O+ defines days on which infections were predicted to be present and DM lesions appeared on leaves.

In a third analysis, a binary logistic regression (or logit model) was used to predict the odds of having a DM infection (the binary dependent variable, *Y*) based on the cumulative values of SEV (the continuous independent variable, *X*) in the following form: P(Y) = 1/(1+exp(−(B0+B1×X))). The odds are defined as the probability that a particular cumulative SEV value results in an infection divided by the probability that it does not result in an infection. Three logit models were fit in which the binary variable *Y* was set to 0 or 1 based on different thresholds of NLL as follows: NLL > 0, >2, and >5 lesions per leaf piece (8 cm^2^). These thresholds were defined based on the data distribution (see Results section). All possible combinations of observed vs. predicted data were organised in a two-by-two contingency table, and were subjected to a Bayesian analysis (Yuen and Hughes, 2002) in order to calculate sensitivity, specificity, and accuracy of the model predictions.

The relationships between the numbers of sporangia from the spore sampler (SPT), as ln(SPT+1), and the variable D”, and between the cumulative SEV and NLL, as ln(NLL+1), were assessed by determining Pearson’s correlation coefficients.

All statistical analyses were carried out using SPSS software (IBM SPSS Statistics, version 25).

## Results

### Weather Conditions

**Figure 4:**
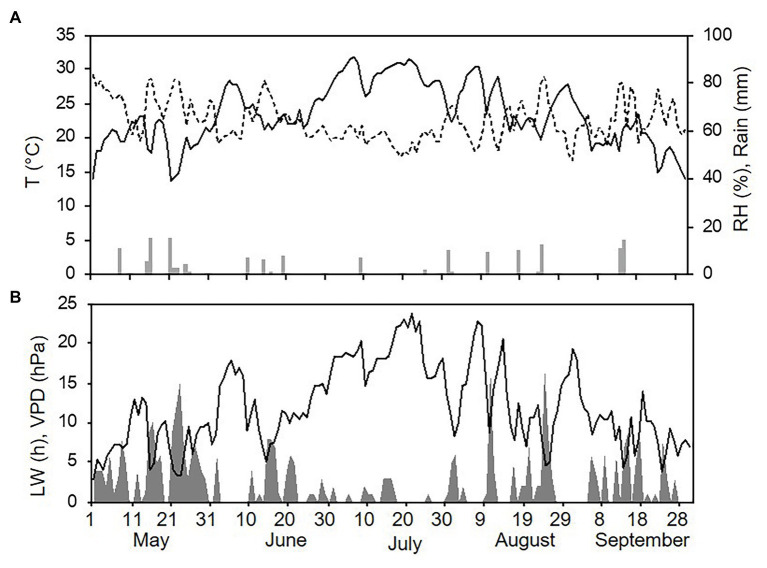
Weather conditions in 2015 during spore sampling periods in the vineyard. **(A)** Air temperature (T, °C, full line), relative humidity (RH, %, dotted line), and rainfall (mm, grey bars). **(B)** Leaf wetness (LW, hours, grey area) and VPD (hPa, full line).

**Figure 5:**
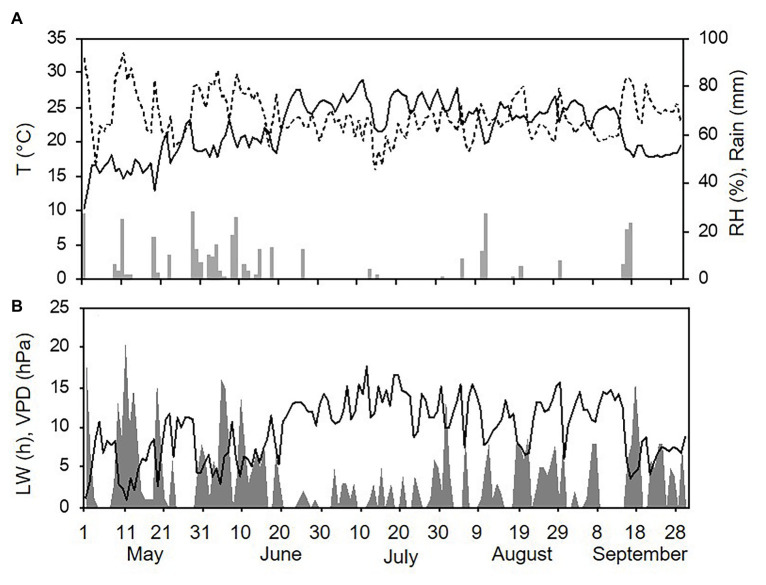
Weather conditions in 2016 during spore sampling periods in the vineyard. **(A)** Air temperature (T, °C, full line), relative humidity (RH, %, dotted line), and rainfall (mm, grey bars). **(B)** Leaf wetness (LW, hours, grey area) and VPD (hPa, full line).

**Figure 6:**
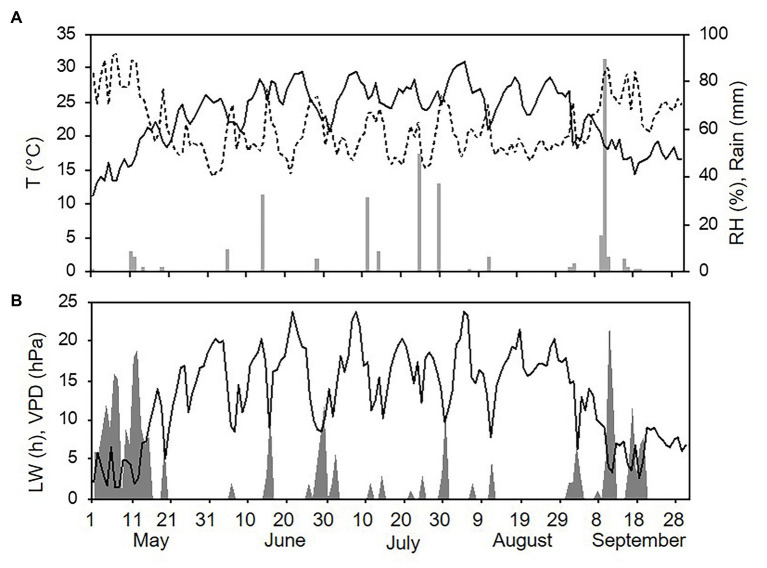
Weather conditions in 2017 during spore sampling periods in the vineyard. **(A)** Air temperature (T, °C, full line), relative humidity (RH, %, dotted line), and rainfall (mm, grey bars). **(B)** Leaf wetness (LW, hours, grey area) and VPD (hPa, full line).

Weather conditions differed among the three sampling years (with one season per year, from 1 May to 30 September). The hottest and driest year was 2015 with an overall average daily temperature of 24.4°C, a minimum (min) of 13.6°C, and a maximum (max) of 31.7°C. The average daily RH was 63%, and total rainfall during the spore sampling season was 155 mm, which was distributed over 32 rainy days (Figure 4A). A total of 295 h of leaf wetness and an average VPD = 12.12 were registered (Figure 4B). The coolest and wettest year was 2016, and May and June in particular were characterised by frequent and intense rain, with prolonged wet periods. Total rainfall was about two times higher in 2016 than in 2015 (364 mm of rain on 43 rainy days in 2016), and the average RH was 68%. The average temperature was 22.4°C (min = 12.9°C and max = 28.8°C; Figure 5A). A total of 499 h of leaf wetness and an average VPD = 9.82 were registered in 2016 (Figure 5B). The 2017 season was quite dry with 314 mm of rain on only 25 rainy days and an average RH = 58%. The average temperature was 23.9°C (min = 14.4°C and max = 30.8°C; Figure 6A). In 2017, there were only 177 h of leaf wetness and an average VPD = 13.25 (Figure 6B).

### Evaluation of the Sporulation Compartment

**Figure 7:**
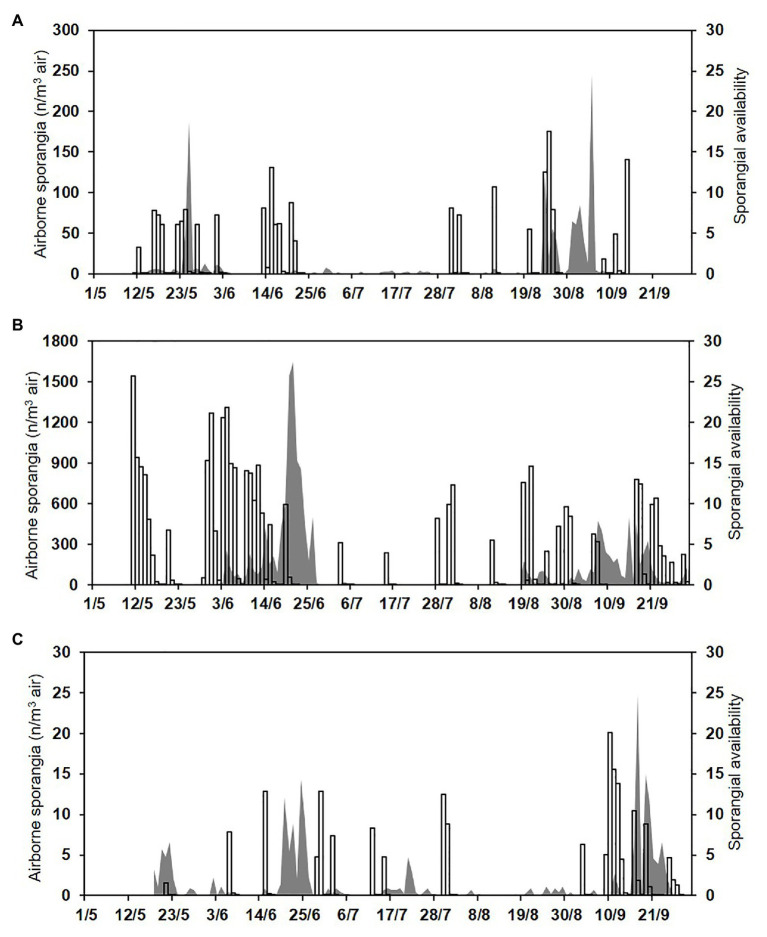
Numbers of the airborne sporangia of *P. viticola* sampled by the spore trapper (SPT; n/m^3^ air, grey area) and sporangial availability predicted by the model (bars) during **(A)** 2015, **(B)** 2016, and **(C)** 2017.

Numbers of the airborne sporangia of *P. viticola* collected by the spore sampler reflected the weather conditions, i.e., they were higher in 2016 than in 2015 or 2017 (Figure 7). In 2015, a total of 1,230 airborne sporangia were detected on 116 of the 127 days of the sampling period (91% of the days). On most days in 2015, however, fewer than 10 sporangia m^−3^ air day^−1^ were detected, but peaks occurred on 25 May (188 sporangia m^−3^ air), 24 August (125 sporangia m^−3^ air), and 5 September (246 sporangia m^−3^ air; Figure 7A). In 2016, a total of 14,485 airborne sporangia were detected on 122 of the 143 days of the sampling period (85% of the days). There were several peaks in 2016 (Figure 7B). In 2017, a total of 193 airborne sporangia were detected; airborne sporangia were detected on only 72 of the 134 days of the sampling period (54% of the days), and there were no peaks (Figure 7C).

Over the total study period, sporangia were, therefore, present in the vineyard air on 310 of the 404 days (76.7% of the days); significant numbers were present on 276 days (68.3% of the days; Figure 7). The model predicted the presence of viable sporangia on 290 days and the absence of sporangia on 114 days. On 266 days, predictions concerning the presence of significant numbers of sporangia were correct, with TPP = 0.78 and TNP = 0.41, giving an overall model accuracy of 0.66 (Table 3); on the remaining 138 days, the model predictions were wrong, with FNP = 0.22 and FPP = 0.59. Based on these data, the posterior probability that there were sporangia when predicted by the model was *P*(P+O+) = 0.74, and the posterior probability that there were sporangia when not predicted by the model was *P*(P−O+) = 0.33 (Table 3). Therefore, there were 62 of 404 days (15.3% of the days) in which sporangia were present but had not been predicted in the vineyard. However, the sporangia sampled on these days represented only 2.9% of the total sporangia sampled in the study period (a total of 461 sporangia of 15,907), and only one of 40 peaks was not predicted, with 246 sporangia m^−3^ air on 5 September 2015.

**Table 3:** Comparison between the presence of *P. viticola* sporangia and infection as predicted by the model (P) and observed in the vineyard (O).

		P (+)^1^	P (−)^1^	Total	Prior probability	Posterior probability		Accuracy
Presence of sporangia	O (+)^2^	214 ^3^TPP = 0.78	62 ^4^FNP = 0.22	276	P(O+) = 0.68	P(P+O+) = 0.74	P(P−O+) = 0.33	0.18^7^
	O (−)^2^	76 ^5^FPP = 0.59	52 ^6^TNP = 0.41	128	P(O−) = 0.32	P(P+O−) = 0.26	P(P−O−) = 0.67	0.66^8^
	Total	290	114	404				
Occurrence of infection	O (+)	37 TPP = 0.80	9 FNP = 0.20	46	P(O+) = 0.43	P(P+O+) = 0.76	P(P−O+) = 0.13	0.61
	O (−)	12 FPP = 0.19	50 TNP = 0.81	62	P(O−) = 0.57	P(P+O−) = 0.24	P(P−O−) = 0.87	0.81
	Total	49	59	108				

1 P+ and P− denote cases in which the model has predicted the presence or absence of viable sporangia, respectively, and cases in which the model has predicted the occurrence or absence of infection, respectively.

2 O+ and O− denote cases in which significant numbers of airborne sporangia were or were not sampled by a volumetric spore sampler, respectively, and cases in which DM lesions were or were not found on grape leaves, respectively.

3 TPP, true positive proportion.

4 FNP, false negative proportion.

5 FPP, false positive proportion.

6 TNP, true negative proportion.

7 Jouden index: TPP+TNP-1.

8 Overall accuracy: number of correct prediction (O+P+ and O−P−)/total cases.

**Figure 8:**
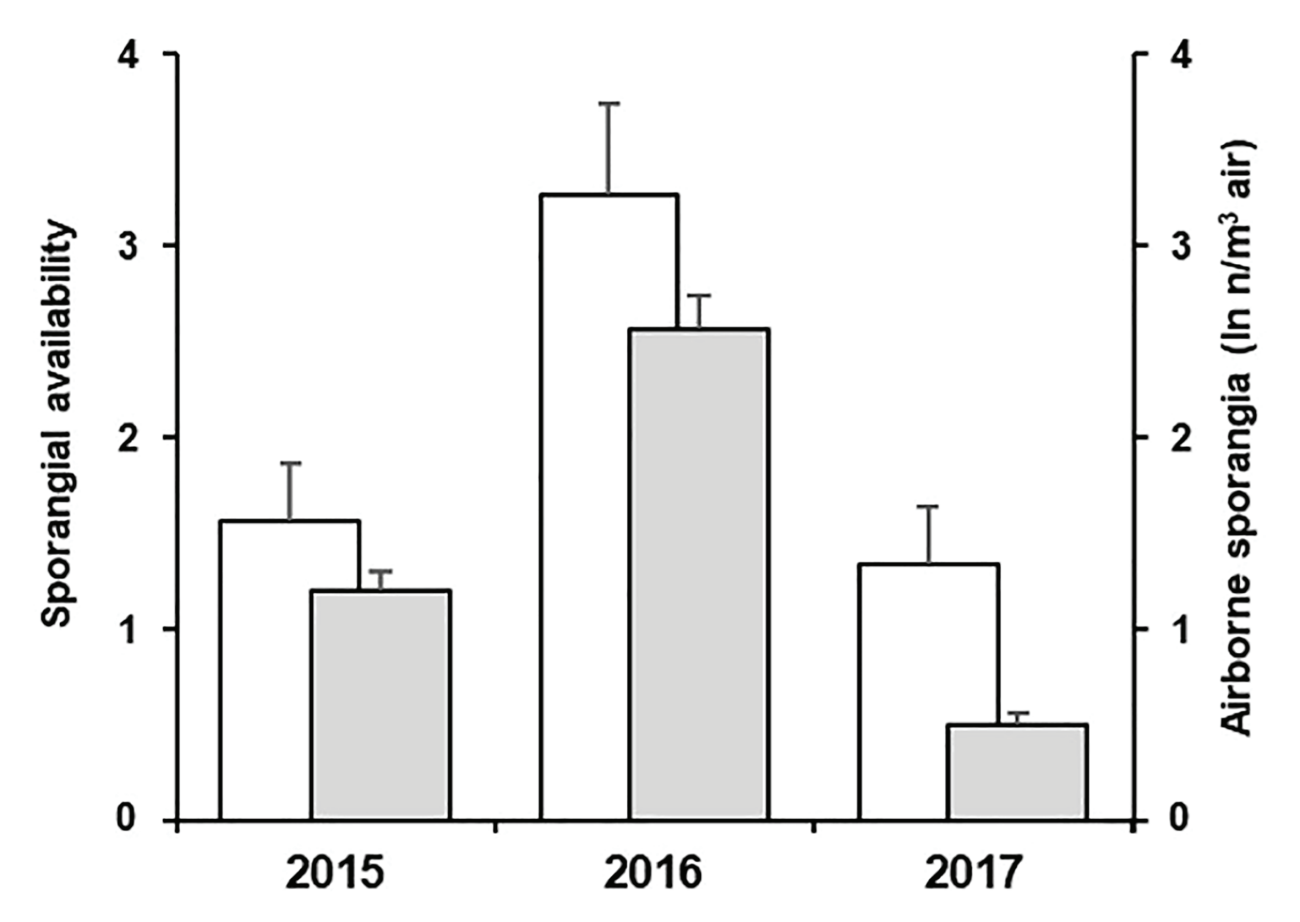
Average values of sporangial availability predicted by the model (white bars) and average ln-transformed values of airborne sporangia (n/m^3^ air) observed (grey bars) in the 3-year experiment. Whiskers represent SEs.

Numbers of sporangia from the spore sampler were correlated with the variable D” of the model, i.e., the availability of viable sporangia, with *r* = 0.255 and *p* < 0.001. In addition, the model was able to account for the among-years difference in the abundance of sporangia (Figure 8). The low correlation coefficient (*r* = 0.255) should be analysed, considering that the model predicts the presence of viable sporangia while the counts from the spore sampler include both viable and non-viable; it follows that some of the sporangia captured by the sampler may not be viable.

### Evaluation of the Infection Compartment

**Figure 9:**
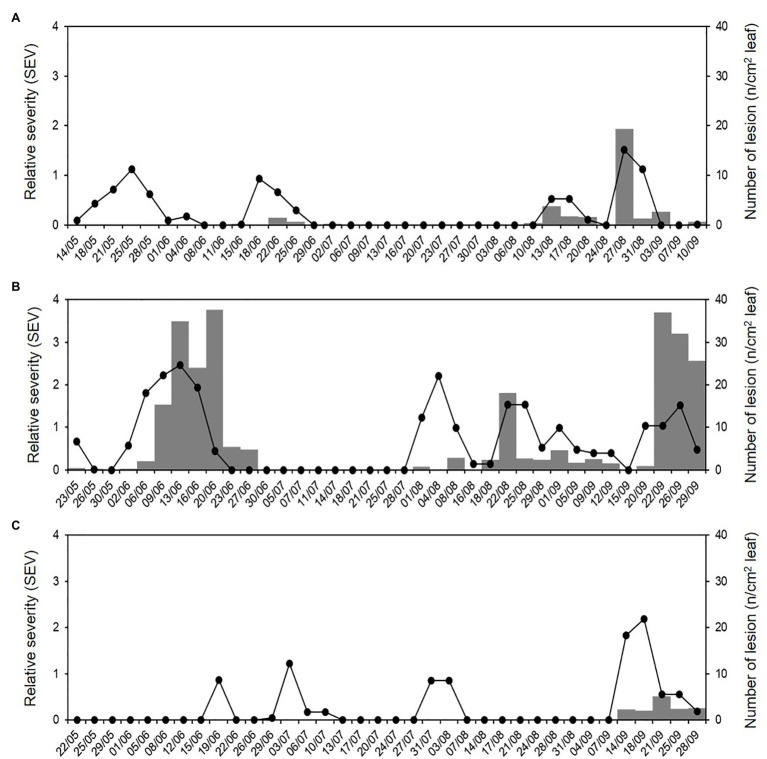
Relative severity of infection (SEV) predicted by the model (line) and number of DM lesions (NLL) on grape leaf fragments (8 cm^2^; grey bars) that were sampled in the vineyard during **(A)** 2015, **(B)** 2016, and **(C)** 2017.

In 46 of 108 cases, the asymptomatic leaf fragments taken from the vineyard showed typical DM lesions after incubation under favourable conditions in the laboratory, indicating that they held latent infections of *P. viticola* at the time of sampling; therefore, occurrence of *P. viticola* infection was observed on 42.6% of the sampling days. The average number of DM lesions (NLL) on these leaf fragments (8 cm^2^ wide) ranged from 0.05 to 37.60 (Figure 9); the main percentiles of the distribution of NLL data were 25^th^ = 0.6, 50^th^ = 2.2, 75^th^ = 4.8, 90^th^ = 27.5, and 95^th^ = 36.3, which showed a relevant positive skewness (1.92) and kurtosis (2.41). The use of the 50^th^ and 75^th^ as thresholds for distinguishing between intermediate and severe DM infections, gave that intermediate and severe infections were observed in 23 (21.3%) and 11 (10.2%) of the 108 sampling days, respectively.

**Figure 10:**
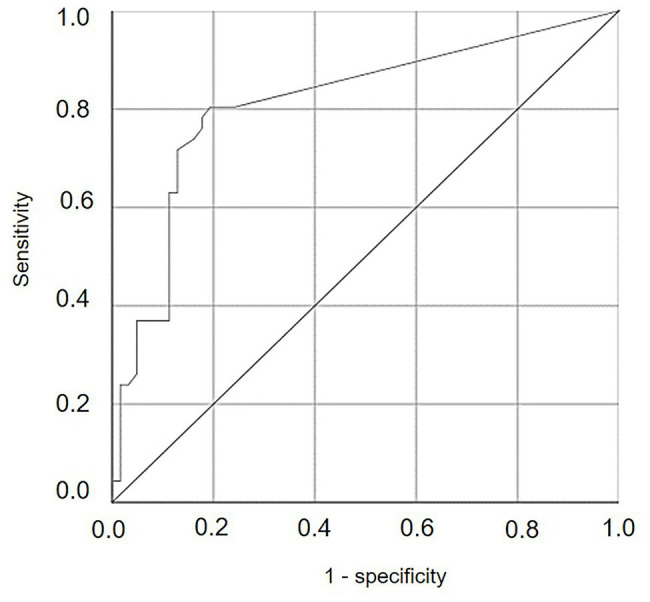
Receiver operating characteristic (ROC) curve showing the trade-offs between sensitivity and specificity for different cut-off values of cumulative values of relative serenity of infection (SEV) calculated by the model for predicting the occurrence of infection on leaves. The diagonal is the line of no-discrimination.

Numbers of DM lesions on leaves were significantly correlated with the cumulative values of SEV calculated by the model, i.e., the relative severity of infections, with *r* = 0.599 and *p* < 0.001. The ROC curve (Figure 10) generated by using different cut-off values of cumulative SEV for predicting the occurrence of infection on leaves was significantly different from the line of no-discrimination (the diagonal line in Figure 10) with *p* < 0.001 and AUROC = 0.814 ± 0.044, indicating that the model predictions were related to the binary prediction of infection. The optimal cut-off point was 0.065, for which sensitivity = 0.804, 1-specificity = 0.194, and *d*^2^ = 0.076.

The use of this cut-off point for determining the number of sampling days on which the model predicted infection (P+) or no infection (P−) provided an overall accuracy = 0.81, sensitivity = 0.80, and specificity = 0.81 (Table 3). The likelihood ratio LR+ [= sensitivity / (1 - specificity)] was 4.16, and the likelihood ratio LR- [= (1 - sensitivity) /specificity)] was 0.24. The posterior probability of correctly predicting no infection was P(P−O−) = 0.873, and the probability of missing a real infection was P(P−O+) = 0.127. Because the prior probabilities were P(O+) = 0.426 and P(O−) = 0.574, the use of model output made it possible to correctly predict infection and, to an even greater degree, to correctly predict no infection. Therefore, the classifier based on the model, specifically based on SEV, performed better for negative than for positive predictions of *P. viticola* infection. Nevertheless, nine cases with real infection were missed by the classifier; these missed infections, however, involved only 14 DM lesions, which represented only 4.4% of the total number of lesions observed (308 lesions).

**Figure 11:**
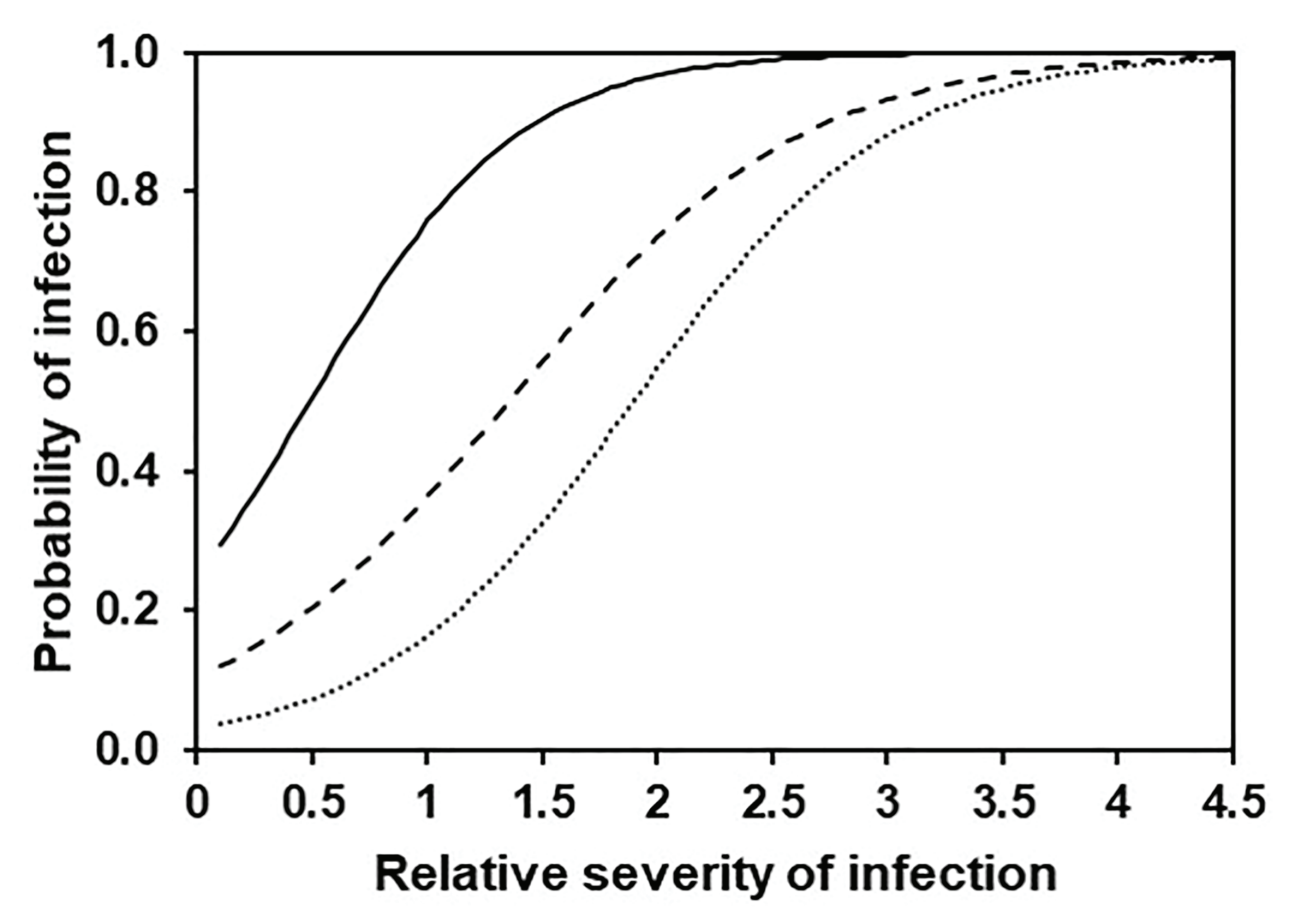
Probability of any (full line), intermediate (dashed line), or severe (dotted line) DM infection on grape leaves according to the binary logistic regression described in Table 4.

The binary logistic regression described relationships between model output and observed infection of grape leaves in the vineyard as a binary outcome (yes, O+; or no, O−) for three levels of disease severity, i.e., the occurrence of any infection (i.e., NLL > 0), a moderate infection (NLL > 2.2), or a severe infection (NLL > 4.8); thresholds for moderate and severe infections (i.e., NLL = 2.2 and 4.8 lesions/8 cm^2^ of leaf) were selected as the values defining the 50^th^ and 75^th^ percentiles of the observed NLL distribution, respectively (Table 4 and Figure 11). This means that, for example, with a predicted SEV of 0.5, the probability of having any infection, an intermediate infection, or a severe infection decreases from P(O+) = 0.505 to 0.206 and 0.073, respectively, i.e., the infection would likely be mild; with a predicted SEV of 2.0, the probability of any infection, an intermediate infection, or a severe infection decreases from P(O+) = 0.967 to 0.735 and 0.546, respectively, i.e., the infection would likely be severe.

**Table 4:** Coefficients and statistics of the binary logistic regression for predicting *P. viticola* infection of grape leaves as a function of the model output relative severity of infection (SEV).

Infection severity^1^	Coefficient^2^	S.E.^3^	Wald chi-square^4^	*p*^5^	Exp(B)^6^	Accuracy^7^
NLL > 0	B1 = 2.234	0.538	17.2	<0.001	9.340	0.75
	B0 = −1.095	0.269	16.5	<0.001	0.335	
NLL > 2.2	B1 = 1.581	0.397	15.9	<0.001	4.861	0.83
	B0 = −2.142	0.354	36.6	<0.001	0.117	
NLL > 4.8	B1 = 1.814	0.47	14.9	<0.001	6.137	0.91
	B0 = −3.442	0.578	35.5	<0.001	0.032	

1 Infection severity for predicted infection is based on the number of DM lesions (NLL) observed on grape leaf fragments (8 cm^2^ wide).

2 Coefficients of the logistic regression in the following form: *P*(Y) = 1/(1+exp(−(B0+B1×X))), expressed in log-odds units, where *P*(Y) is the probability of infection, and X is the predicted severity SEV.

3 Standard error of coefficients.

4 Wald chi-square value.

5 Two-tailed value of *p* used in testing the null hypothesis that the coefficient is 0.

6 Odds ratios for the coefficients (predictors) as the ratio of the probability of success in predicting infection over the probability of failure.

7 Overall accuracy of the model expressed as the proportion of cases that were correctly classified by using the equation.

## Discussion

In the present work, a mechanistic model was developed to predict the occurrence and severity of secondary infections of *P. viticola* on grapevines based on environmental conditions. The model is weather-driven and involves the main processes of the secondary infection cycles in the grapevine-DM pathosystem, i.e., sporulation, dispersal and deposition, and infection. These processes were quantitatively represented by previously developed equations (Lalancette et al., 1988b; Magarey et al., 2005; Brischetto et al., 2020) and recent research findings (Kennelly et al., 2007; Caffi et al., 2013a). The model also considers equations accounting for the mortality of sporangia as used in Brischetto et al. (2020); to our knowledge, this is the first model that explicitly considers the survival of *P. viticola* sporangia. Only a few of the models that have been developed for predicting secondary infections by *P. viticola* account for the survival of sporangia. In developing the PLASMO model, Orlandini et al. (2008) included an equation for the survival of sporangia (Rosa et al., 1993) as a function of temperature and relative humidity as reported by Blaeser and Weltzien (1978); the parameters of the equation, however, were not specified, which precludes the use of the equation by other researchers. Equations for the survival of sporangia were also mentioned in VitiMeteoPlasmopara (Bleyer et al., 2008) and RIMpro-Plasmopara (Trapman, 2013), but these equations were not explicitly described.

The model developed in the current research was evaluated against the data collected in a 3-year vineyard study of the dynamics of the airborne *P. viticola* sporangia and the occurrence of infection on leaves exposed to that inoculum (Brischetto et al., 2020). These data were not used in model building and represented different conditions of temperature, humidity, rain, and leaf wetness duration, as shown in Figure 3. Therefore, the evaluation can be considered robust (Camase, 1996; Pascual et al., 2003; Rossi et al., 2010).

Concerning airborne *P. viticola* sporangia, the current study confirmed other reports that *P. viticola* sporangia are a common component of the airborne microflora of vineyards (Diaz et al., 1998; Albelda et al., 2005; Fernandez-Gonzalez et al., 2009; Magyar et al., 2009; Fernández-González et al., 2011, 2019; Martínez-Bracero et al., 2019; Rodríguez et al., 2020). This is highly relevant to DM management: while the inoculum for primary infection is available only following a rain, the secondary inoculum is always present when *P. viticola* is established in the vineyard.

Even though *P. viticola* sporangia were frequently sampled from the air of the DM-affected vineyard in the current study, their concentrations changed within and among seasons. The model was able to interpret this variability with an overall accuracy of 0.66 over 1 (whit 1 over 1 would indicating the perfect agreement), which is quite low. The low model performance has two main explanations. First, the model predicts the sporulation dynamics for a DM lesion (or a cohort of DM lesions that is coeval) but does not consider the number of DM lesions that are present in the vineyard; the spore sampler data, in contrast, are obviously affected by the number of sporulating DM lesions. Second, the model predicts the presence of viable sporangia while the spore sampler, as noted earlier, collects both viable and non-viable sporangia, which cannot be distinguished in spore counts. The real number of viable sporangia was not considered in this study because its quantification is difficult, i.e., it requires the collecting of sporangia and the testing of their viability by inoculating susceptible grape tissue, by staining sporangia with fluorochromes (Sergeeva et al., 2002), or by using a spectrophotometer (Hong and Scherm, 2020). Molecular methods have been developed for testing the viability of fungal spores (Vesper et al., 2008; Cangelosi and Meschke, 2014; Al-Daoud et al., 2017; Vilanova et al., 2017), but they have not been tested with *P. viticola*. For practical purposes, i.e., for improving DM control, the model should be evaluated for its ability to predict periods with no sporangia (i.e., for negative prognosis) and periods with peaks of sporangia; if reasonably correct, these predictions would enable growers to identify periods with no/low risk or high risk, respectively. When used for negative prognosis, the model made it possible to increase the probability to predict no sporangia by two times compared to the prior probability, which were P(P−O−) = 0.67 and P(O−) = 0.32, respectively. In addition, fewer than 3% of the total sporangia found in this study were sampled when not predicted by the model, and this confirmed that the model may be useful when used for negative prognosis. When the model was used for predicting peaks of sporangia, only one of 40 peaks was unpredicted; this occurred on 5 September 2015, when there were only 4 h in the dark, a maximum RH of 75%, no leaf wetness, and an average temperature of 19°C. Based on the current knowledge, these conditions are not conducive for production of sporangia by *P. viticola*; therefore, the presence of these sporangia in the vineyard was unclear and not predictable. Our current understanding is that the sporulation of *P. viticola* is inhibited by light and occurs at night (Muller and Sleumer, 1934; Yarwood, 1937; Rumbolz et al., 2002), and is triggered in darkness by a period of at least 3 h with RH ≥80%, rain, or leaf wetness (Caffi et al., 2013a).

Concerning the *P. viticola* infection on leaves, the comparison between model prediction and reality provided an overall accuracy of 0.81, with true proportions >0.8 for both positive and negative proportions. Overall, the results showed that the model was a useful predictor of infection and especially for negative prognosis, because the posterior probability for the infection not to occur when not predicted was 0.87. When used for negative prognosis, the model missed some infections (exactly 9 of 108), but these infections were mild and accounted for only 4.4% of the total DM lesions observed in the study.

Negative prognosis may be very useful for controlling the disease during its secondary spread. The control of DM after fruit set, when secondary infections are dominant, is usually based on calendar applications (Gessler et al., 2011), with fungicides applied every 7–10 days depending on the fungicide used, weather and especially rainy conditions, the growth of new leaves from lateral shoots (Caffi and Rossi, 2018), and plant growth in relation to ontogenic resistance of clusters (Kennelly et al., 2001; Gadoury, 2015). In such a situation, a model able to identify periods in which the DM risk is nil or very low may be helpful for avoiding fungicide interventions when not needed or for lengthening the interval between two sprays. In other words, the model could help growers move from calendar-based to risk-based fungicide schedules.

Like the model developed by Caffi et al. (2016), the model in the current study was developed with infection-of-leaves data and was validated by comparing model predictions with leaf infection in a vineyard. DM epidemics, however, are dual epidemics, i.e., they develop on two main organs, leaves and clusters, in the course of a cropping season (Savary et al., 2009). Grape rachises and berries are susceptible to *P. viticola* from inflorescence emergence to when they become resistant because of ontogenic resistance. Ontogenic or age-related resistance results from the loss of infection courts due to the conversion of stomata into lenticels and to the clogging of stomatal openings (Gindro et al., 2012; Fröbel and Zyprian, 2019); this occurs between 1 and 6 weeks post-bloom (Kennelly et al., 2005). Like DM lesions on leaves, DM lesions on berries and rachises produce secondary sporangia until the stomata lose functionality and do not further support sporulation; these sporangia may also contribute to the disease.

Models for DM developed in the past (Lalancette et al., 1988b; Blaise and Gessler, 1990; Hill, 1990; Magarey et al., 1991; Magnien et al., 1991; Orlandini et al., 1993; Ellis et al., 1994; Blaise et al., 1999; Leroy et al., 2013) did not account for the infection of clusters and for their contribution to the epidemic *via* production of sporangia, with the exception of the model of Bove et al. (2020a). The latter model includes the role of clusters in the DM epidemic through a transmission coefficient that numerically links the two components, leaves, and clusters; that coefficient enables the model to simulate the level of cluster infection based on DM severity on the foliage at successive crop stages (Savary et al., 2009). Weather-driven models for DM epidemics developed to date have failed to explicitly consider infection of clusters because there are no published data on how infection of clusters is affected by temperature, wetness duration, and infection or disease severity. Research is, therefore, needed so that existing models can be modified to account for the dual nature of *P. viticola* epidemics.

As indicated in the previous paragraph, there are possible weaknesses in using leaf-based infection models to guide fungicide applications for protection of both leaves and clusters. Caffi et al. (2011), however, demonstrated that the scheduling of fungicide applications based on a model for primary infection of leaves (Caffi et al., 2009) efficiently controlled DM on both leaves and clusters. This would also likely be true for the present model for secondary infections, given that secondary infection cycles are more important than primary cycles late in the season, when ontogenic resistance greatly reduces the susceptibility of clusters to *P. viticola*, such that the disease develops only on leaves.

Based on the results of the current research, the model described and tested here could be used to advise growers about the risk of secondary DM infections; this would enable growers to make informed decisions about crop protection, in compliance with the general rules of IPM and of Directive 2009/128/CE on the sustainable use of pesticides (Rossi et al., 2012). Future field experiments may be useful for demonstrating the advantages of model-timed sprays vs. intensive calendar-based spray programs.

## Data Availability Statement

The raw data supporting the conclusions of this article will be made available by the authors, without undue reservation.

## Author Contributions

VR mainly contributed to the conception and the design of the study. FB and CB collected field data. All authors contributed to the analysis of results and collaborated in writing the manuscript. All authors contributed to the article and approved the submitted version.

### Conflict of Interest

FB was employed by Horta srl.

The remaining authors declare that the research was conducted in the absence of any commercial or financial relationships that could be construed as a potential conflict of interest.
